# Seasonal Variation and Sexual Dimorphism of the Microbiota in Wild Blue Sheep (*Pseudois nayaur*)

**DOI:** 10.3389/fmicb.2020.01260

**Published:** 2020-06-26

**Authors:** Zhaoling Zhu, Yewen Sun, Feng Zhu, Zhensheng Liu, Ruliang Pan, Liwei Teng, Songtao Guo

**Affiliations:** ^1^College of Wildlife and Protected Area, Key Laboratory of Conservation Biology, State Forestry Administration, Northeast Forestry University, Harbin, China; ^2^College of Economics and Management, Jiamusi University, Jiamusi, China; ^3^Shaanxi Key Laboratory for Animal Conservation, Northwest University, Xi’an, China; ^4^School of Human Sciences, The University of Western Australia, Perth, WA, United States; ^5^International Centre of Biodiversity and Primate Conservation, Dali University, Dali, China

**Keywords:** wild blue sheep, gut microbiota, seasonal and sexual variations, short-chain fatty acids, aggregation

## Abstract

Microbiota of the wild blue sheep (*Pseudois nayaur*) presents a seasonal variation due to different dietary selection and feeding strategies from different ecological niches chosen by different sex in summer. To address those issues, we analyzed the variation of gut microbiota based on the material from the feces, with 16S rRNA and meta-genome aimed to explore seasonal and gender differences. The results indicate that seasonal dietary changes and gender differentiation, as expected, cause the variation in sheep’s gut microbiota structure. The variation of the former is more significant than the latter. Dominant Firmicutes exists a significantly higher abundance in summer than that in winter. Subordinate Bacteroides expresses no seasonal difference between the two seasons. Compared with the winter group, the summer group is featured by abundant enzymes digesting cellulose and generating short-chain fatty acids (SCFAs), such as beta-glucosidase (EC: 3.2.1.21) for cellulose digestion, and butyrate kinase (EC:2.7.2.7) in butyrate metabolism, implying that the changes of the composition in intestinal flora allow the sheep to adapt to the seasonalized dietary selection through alternated microbial functions to reach the goal of facilitating the efficiency of energy harvesting. The results also show that the blue sheep expresses a prominent sexual dimorphism in the components of gut microbiota, indicating that the two sexes have different adaptations to the dietary selection, and demands for physical and psychological purposes. Thus, this study provides an example of demonstrating the principles and regulations of natural selection and environmental adaptation.

## Introduction

The community of the gut microbiota is very important to maintain animals’ dynamic stability of the gastrointestinal tract and help the hosts adapt to alternative dietary choices (Amato et al., 2015). The gut microbiota, however, always changes in responding to the alterations of food resources and seasonal variation (Hooper et al., 2012; Morgan et al., 2012; Markle et al., 2013). Thus, understanding such association, particularly the mechanism of how specific gut microbes respond to dietary selection, allows us to amend the formed conservation strategies and tactics more scientifically for the animals studied (Bergmann et al., 2015; Kartzinel et al., 2019). Some recent studies indicate that seasonal reconfiguration of the microbiota in response to the dietary fluctuation exists in the Hadza hunter-gatherers in Tanzania (Smits et al., 2017), the western lowland gorillas and chimpanzees (Hicks et al., 2018), and red squirrels (Ren et al., 2017). This implies that seasonal dietary change leads to the reconfiguration of hosts’ gut microbes. It is reported that there also exist individual differences in gut microbiota, particularly gender differences that may be prominent by the dietary selection, caused by alternative demands of different sexes during a non-breeding period (Bowyer, 2004; Ruckstuhl, 2007). Sexual dimorphism may also be related to the seasonal dietary variation that needs to be attested.

Most ruminants highly rely on microbial communities in the gut to digest food components, which also make animals adjust dietary choices according to phenological periods of plants, and physical and psychological demands (Espunyes et al., 2019; Takada and Minami, 2019). It is reported that seasonal variation in food resources can cause the alteration of gut microbial communities (Faith et al., 2013; Ley et al., 2013; Bergmann et al., 2015; Hu et al., 2018), which then affect energy production and social behavior among individuals, particularly between the sexes.

Gregarious animals synchronize their foraging activities and resources; hence, there is less dietary variation among the individuals (Galef and Giraldeau, 2001). Some species whose populations or groups segregate and/or aggregate show great differences in regional and seasonal dietary selection and adaptation, which cause remarkable variation in microbial communities (Ruckstuhl and Neuhaus, 2002; Han et al., 2019; Stone et al., 2019). On the other hand, sexual dimorphism exists in diet and microbial communities (Bowyer, 2004; Ruckstuhl, 2007). This especially applies to the species with a great variety of forage areas and food choices, among them including the blue sheep, *Pseudois nayaur* (Mooring et al., 2003; Bonenfant et al., 2004). This species has a social behavior in which males separate from females in summer and autumn, but congregate with the females in winter (Li, 2006). Some researchers indicated that male ungulates increase fat accumulation by extending feeding time before the aggregation (Festa-Bianchet et al., 1990; Jönsson, 1997; Yoccoz et al., 2002; Mysterud et al., 2005). They also consume a large amount of energy, resulting in further declining body weight due to frequent copulation during rutting periods in winter (Clutton-Brock et al., 1982; Pérez-Barberia et al., 1998; Pelletier, 2005). However, it is hard to work out how a male’s body weight is reduced, which could be due to the changes of foraging time and periodical resource variation (Ferrari et al., 1998; Mysterud et al., 2005). On the one hand, the dietary alteration can influence gut microbiota and modify microbial function, which probably aggravates the reduction of weight (Muegge et al., 2011; Ley et al., 2013). On the other hand, we do not know whether some social behaviors could influence the variation of gut microbiota. Thus, the studies on the variations of microbial composition and its function can allow us to understand how dietary components are digested and broken down, and explore the clues comprehending animals’ physiological and behaviors profiles, possible energy acquisition, the choice of reproductive sites, and different foraging strategies between non-breeding and breeding periods (Shabat et al., 2016).

The blue sheep is a model for such an endeavor. This species is distributed in central Asia, including western mountains in China. Its populations are found in an altitudinal range between 2000 and 6000 m, with the habitats of alpine floral structure in the northern temperate zone showing distinct seasonal vegetation variation (Di, 1987). The species consumes diverse vegetables, including herbs, forbs, shrubs, and trees, and takes alternative plant parts, such as stems, leaves, flowers, fruits, and barks (Liu et al., 2007). Moreover, our previous findings suggest that this species exhibits remarkable seasonal dietary variation (Liu et al., 2007; Chang, 2010). Socially, they form groups in moving, including gender-oriented or mixed ones in different seasons (Cao et al., 2005; Zhang et al., 2006).

This study, aimed at analyzing the relationship between seasonal social behavior and gut microbiota, needs to address some biological issues: (1) whether there is a significant sexual dimorphism in gut microbiota; (2) whether a seasonal dietary change can cause a significant difference in gut microbiota; (3) whether seasonal variation of the diets results in significant differences in the microbial function in the gut, which may have caused prominent influence on digestion and energy absorption, particularly the males.

## Experimental Procedures

### Fecal Sample Collection

**FIGURE 1:**
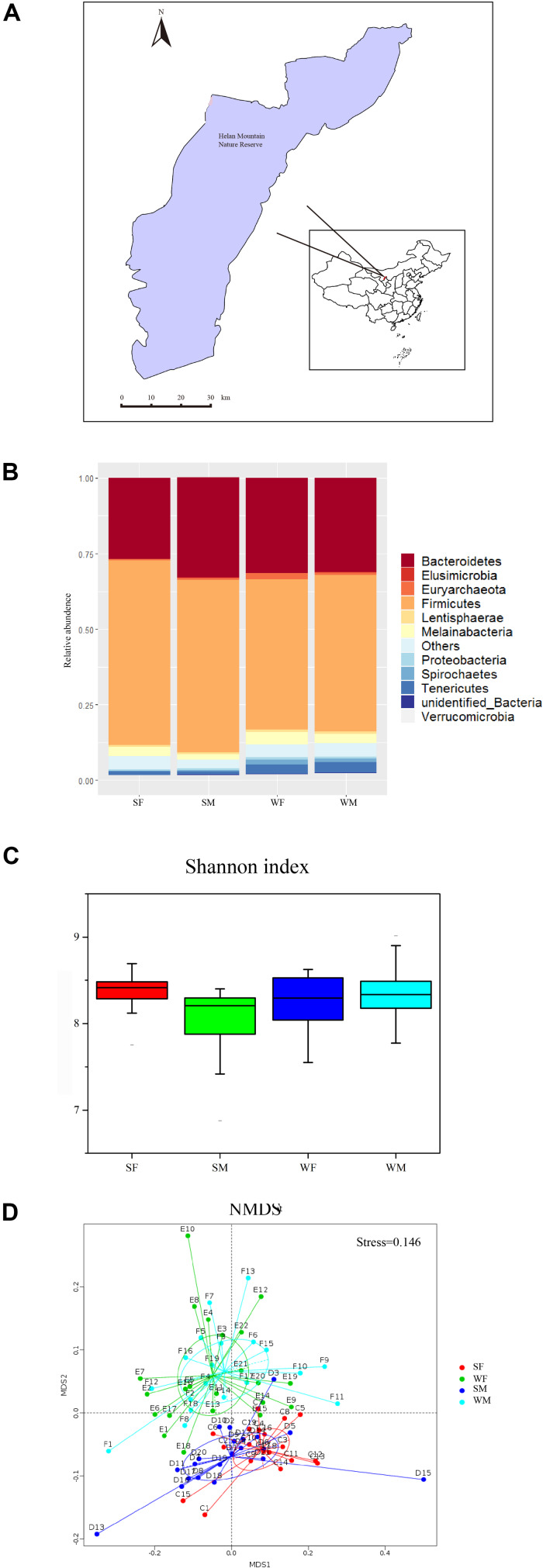
The geographic location, and gut microbiome composition among groups. **(A)** The geographic location of the sample collection site. All the samples were collected in the Helan Montoin, Yinchuan (N 38° 44’, E106°01’) during July-August and November-December 2017. **(B)** Relative abundence difference among different groups in Phylum level(SF = summer female, SM = summer male, WF = winter female and WM = winter male). **(C)** The Shannon index of the four groups. **(D)** The Non-Metric Multi-Dimensional Scaling (NMDS) based on OTUs of the four groups. Each point in the figure represents a sample, and the distance between the points indicates the degree of difference. The Stress is less than 0.2, which indicates that NMDS can accurately reflect the differences between the samples.

We collected fecal samples with the line transect method in the Helan Mountain (N 38° 44’, E 106° 01’) during July–August and November–December 2017 (Figure 1A). We surveyed seven geomorphological transects covering all vegetation types in which blue sheep inhabit. The length of the transects was from 2 to 6 km (Liu et al., 2009). All the samples were collected with sterilized tweezers by the researchers who wore a mask and disposable PE gloves. Tweezers and gloves were individually used for each of the samples that were maintained in the ice box and then kept in the refrigerator at −80°C for 2 h. We collected a total of 369 fecal samples from which we randomly selected 81 for 16s rRNA analysis. Then, all the samples were sequenced by metagenomics (A1, A2, A7, E3, and E5 in winter, and B1, B2, B7, C18, and C19 in summer) (Supplementary Table 1). All samples were divided into four groups: females in winter (WF, *n* = 22, E1–E22), males in winter (WM, *n* = 19, F1–F19), females in summer (SF, *n* = 19, C1–C19), and males in summer (SM, *n* = 21, D1–D21).

### Individual and Gender Identification

Fecal DNA was extracted using the kits of QIAamp Fast DNA Stool Mini (Qiagen, Handelsregister: Amtsgericht Düsseldorf HRB 45822 USt-IdNr.: DE 121386819) and was detected by 1.0% of agarose gel electrophoresis (AGE). We used 10 pairs of microsatellite primers (FCB48, ILSTS011, BM1329, INRABERN172, SRCRSP3, JMP58, PND01, PND04, PND05, and PND06) (Yang et al., 2015) to carry out individual identification. Gender identification was performed with the enamel gene PCR method (Ennis and Gallagher, 1994; Huber et al., 2002).

### 16S rRNA Amplification

Based on individual and gender identification, fecal samples were grouped by season and gender, separately, for sequencing. CTAB method was used to extract DNA samples, and 1% AGE was used to detect the purity and concentration of the DNA (Jami and Mizrahi, 2012). DNA samples were then diluted to 1 ng/μl with sterile water. Bacterial universal primers 515F (5′-GTGCCAGCMGCCGCGGTAA-3′) and 806R (5′-GGACTACHVGGGTWTCTAAT-3′) were used for the amplification of 16S rRNA V4 region (Caporaso et al., 2011), and all PCR amplifications were performed using New England Biolabs’ Phusion^®^ High-Fidelity PCR Master Mix with GC Buffer and high-fidelity enzyme. PCR reaction system includes 50.0 μl of the total volume, 5 × Phusion HF buffer 10 μl, 10 mM dNTPs 1 μl, template DNA 1.5 μl (150 ng), Phusion DNA Polymerase 0.5 μl, primer 515F 0.5 μM, primer 806R 0.5 μM, and dd H_2_O add to 50 μl. PCR reaction procedure included pre-denaturation at 98°C for 30 s, denaturation at 98°C for 10 s, annealing at 55°C for 30 s, extending at 72°C for 2 min, reacting for 35 cycles, and finally extending at 72°C for 20 min. The PCR products were purified by GeneJETTM Gel Recovery Kit (Thermo Scientific, Waltham, MA, United States).

### 16S rRNA Library Construction and Sequencing

The library was constructed by Ion Plus Fragment Library Kit 48 rxns (Thermo Scientific, Waltham, MA, United States) following the manufacturer’s instructions, and index codes were added. The library quality was assessed using a Qubit@ 2.0 Fluorometer (Thermo Scientific, Waltham, MA, United States). Finally, the library was sequenced on an Ion S5TM XL platform and 400-bp single-end reads were generated.

### Metagenome Library Construction and Sequencing

A total of 1 μg of DNA per sample was used as input material for the DNA sample preparations. Sequencing libraries were generated using the NEBNext^®^ Ultra^TM^ DNA Library Prep Kit for Illumina (NEB, United States) following the manufacturer’s recommendations. Briefly, the DNA sample was fragmented by sonication to a size of 350 bp, and then its fragments were end-polished, A-tailed, and ligated with the full-length adaptor for Illumina sequencing with further PCR amplification. At last, PCR products were purified (AMPure XP system) and libraries were analyzed for size distribution by Agilent2100 Bioanalyzer (Agilent Technologies, United States), and quantified using real-time PCR. After cluster generation, the library preparations were sequenced on an Illumina HiSeq 2500 platform, and paired-end reads were generated.

### Bioinformatic Analysis of 16S rRNA Gene and Metagenome Sequences

Single-end reads from 16S rRNA sequencing were assigned to the samples based on their unique barcode and truncated by cutting off the barcode and primer sequence. Quality filtering on the raw reads was performed under specific filtering conditions to obtain the high-quality clean reads according to the Cutadapt (V1.9.1,^1^) quality-controlled process (Martin, 2011). The reads were compared with the reference database (Silva database,^2^) (Quast et al., 2013) using UCHIME algorithm (UCHIME Algorithm,^3^) (Edgar et al., 2011) to detect and remove the chimera sequences (Haas et al., 2011). Then, the Clean Reads were finally obtained.

Sequence analysis was performed by Uparse software (Uparse v7.0.1001,^4^) (Edgar, 2013). Sequences with ≥97% similarity were assigned to the same OTUs. Representative sequence for each OTU was screened for further annotation. For each representative sequence, the Silva Database^2^ (Quast et al., 2013) was used based on Mothur algorithm to annotate taxonomic information. In order to study the phylogenetic relationship of different OTUs, and the difference of the dominant species in different groups, multiple sequence alignment was conducted using the MUSCLE software (Version 3.8.31,^5^) (Edgar, 2004). OTUs’ abundance information was normalized using a standard of sequence number corresponding to the sample with the least sequences. Subsequent analysis of alpha diversity and beta diversity was performed based on this normalized output data.

Pre-processing the Raw Data obtained from the Illumina HiSeq sequencing platform using Readfq (V8,^6^) was conducted to acquire the Clean Data for subsequent analysis. The specific processing steps are as follows: (a) remove the reads that contain low-quality bases (quality threshold value ≤ 38) above a certain portion (length of 40 bp); (b) remove the reads in which the N base has reached a certain percentage (length of 10 bp); (c) remove the reads sharing the overlap above a certain portion with Adapter (length of 15 bp). Considering the possibility that host pollution may exist in samples, Bowtie2.2.4 software (Bowtie2.2.4,^7^) was used to filter the reads that are of host origin (Karlsson et al., 2012, 2013). The Clean Data was assembled and analyzed (Luo et al., 2012) by SOAPdenovo software (V2.04,^8^) and then interrupted the assembled Scaftigs from N connection and leave the Scaftigs without N (Mende et al., 2012; Nielsen et al., 2014; Qin et al., 2014). All samples’ Clean Data were compared to each Scaffolds respectively by Bowtie2.2.4 software to acquire the PE reads not used (Qin et al., 2014).

MetaGeneMark (V2.10) was used for ORF prediction (Ennis and Gallagher, 1994; Huber et al., 2002; Li et al., 2014; Yang et al., 2015). The redundant prediction result was removed by cd-hit (V4.5.8) (Li and Godzik, 2006). Bowtie2.2.4 (Caporaso et al., 2011) was used to obtain the gene catalog (Unigenes) for subsequent analysis (Huber et al., 2002). As for degree information, software DIAMOND (v0.9.9.110) (Mende et al., 2012) was used to compare the Unigenes with the bacterial, fungi, archaea, and virus sequences extracted from the NR database (Version 2018-01-02) in the NCBI, and to determine species annotation of the sequence. DIAMOND (v0.9.9.110) software was then used to compare Unigenes with KEGG, eggNOG, and CAZy function databases, and select Best Blast Hit results for subsequent analysis (Karlsson et al., 2013; Nielsen et al., 2014).

### Statistical Analysis

Cervus 3.0 software was used for individual identification of the blue sheep (Brookfield, 1996). The diversity index of all the samples was calculated with QIIME software (Version 1.7). The dilution curve (Rarefaction Curve) and Rank abundance curve were drawn by R software (Version 2.15.3). The differences between the groups were analyzed by the non-parametric Wilcox test when the group number was two. Kruskal rank-sum test was used when that number was more than two. A statistically significant level was determined by *p* < 0.05. Anosim and MRPP (Multi Response Permutation Procedure) were utilized to analyze the significant differences of microbial community structure between the groups. LEfSe (LDA Effect Size) software was applied to identify species differences (Segata et al., 2011).

## Results

### Individual and Gender Identification

A total of 282 blue sheep individuals were identified from 369 blue fecal samples, including 152 males and 130 females. In total, 101 alleles were detected in 10 microsatellite loci with an average number of 10.1. The observed mean and expected heterozygosity were 0.7737 and 0.6512, separately. Polymorphic information content (PIC) is 0.6128. Eight microsatellite loci are consistent with the Hardy-Weinberg equilibrium test.

### Taxonomic Differences of the Blue Sheep

Shannon index of the four groups (SF, SM, WF, and WM) shows a significant difference among them (K–S test, chi-squared = 13.64, df = 3, *p* < 0.01) (Figure 1C, Supplementary Figure 1A). SM group has the lowest index (Supplementary Table 1).

Dominant phylum identified is Firmicutes (54.1% of the total), followed by Bacteroides (30.42%) (Supplementary Table 2). The four groups can, however, be clearly separated based on the structure of gut microbiota. There is no gender difference in Firmicutes, while there is a significant seasonal difference: the summer group expresses a significant higher abundance than the winter group (Wilcox test, *n* = 81, *W* = 1248, *p* < 0.01); a significant gender difference in Bacteroides was found in the summer group (Wilcox test, *n* = 39, *W* = 91, *p* < 0.01), and there is no significant seasonal difference between summer and winter groups (Wilcox test, *n* = 81, *W* = 713.5, *p* > 0.05).

In order to test whether there exist significant differences in intestinal microbial communities among the groups, we conducted Adonis analysis (Supplementary Table 3) and Non-Metric Multi-Dimensional Scaling (NMDS) analysis based on OTUs of the four subgroups (SF, SM, WF, and WM) (Figure 1D, Supplementary Figures 1B,C for PCA and PCoA). The results show that the variation due to seasonal difference is more significant than that due to the gender. It can be seen from the figure in which individuals in the same season are remarkably clustered together, and the individuals of different sexes in the same season group do not show a significant separation.

**FIGURE 2:**
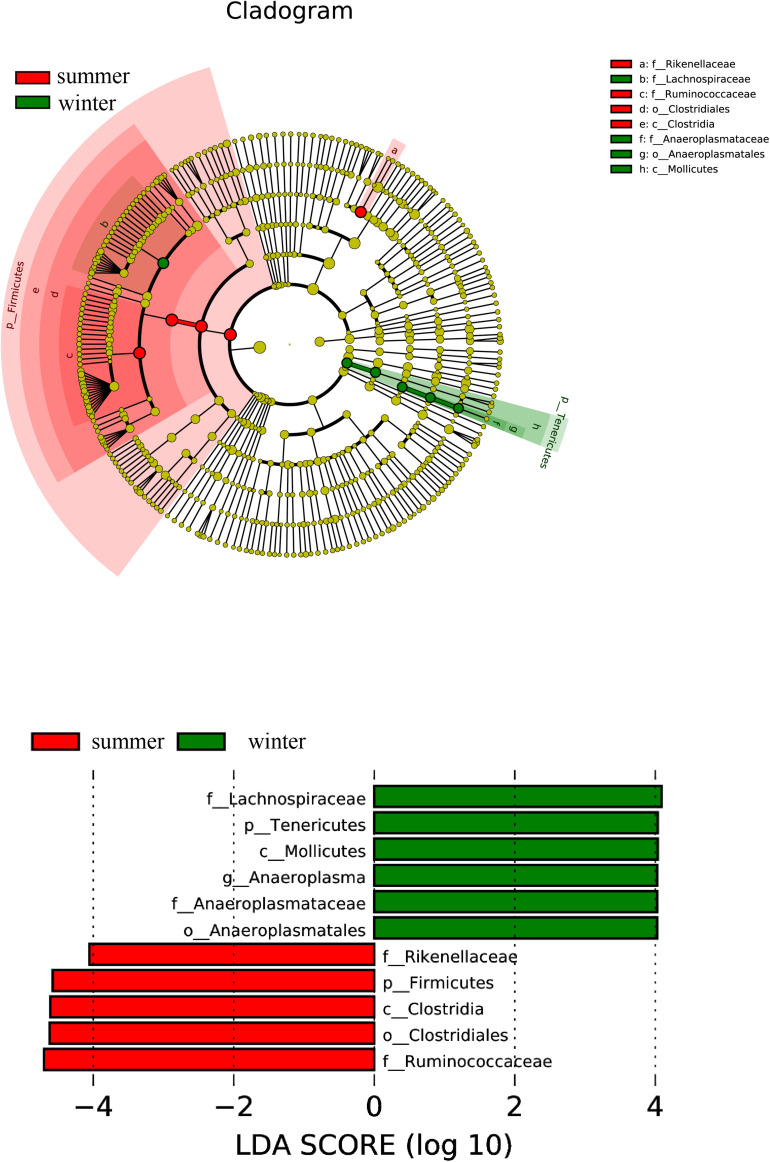
Distribution histogram of LDA and system evolutionary distribution based on biomarkers with statistically significant differences in abundance between the two groups.

To identify differential flora abundance and the associated categories of the intestinal microbe communities, we used an LDA Effect Size (LEfSe) analysis (Score >4) to seek biomarkers showing significant differences between the groups (Figure 2). The results indicate that biomarkers in the summer group are Firmicutes, Rikenellaceae, Ruminococcaceae, Clostrida, and Clostridium, which belong to Firmicutes, except for Rikenellaceae belonging to the Bacteroidetes. The biomarkers in the winter group can be categorized into the Tenericutes, except for Lachnospiraceae.

### Differences in Metabolic Pathways of Microbes Between Two Seasons

**FIGURE 3:**
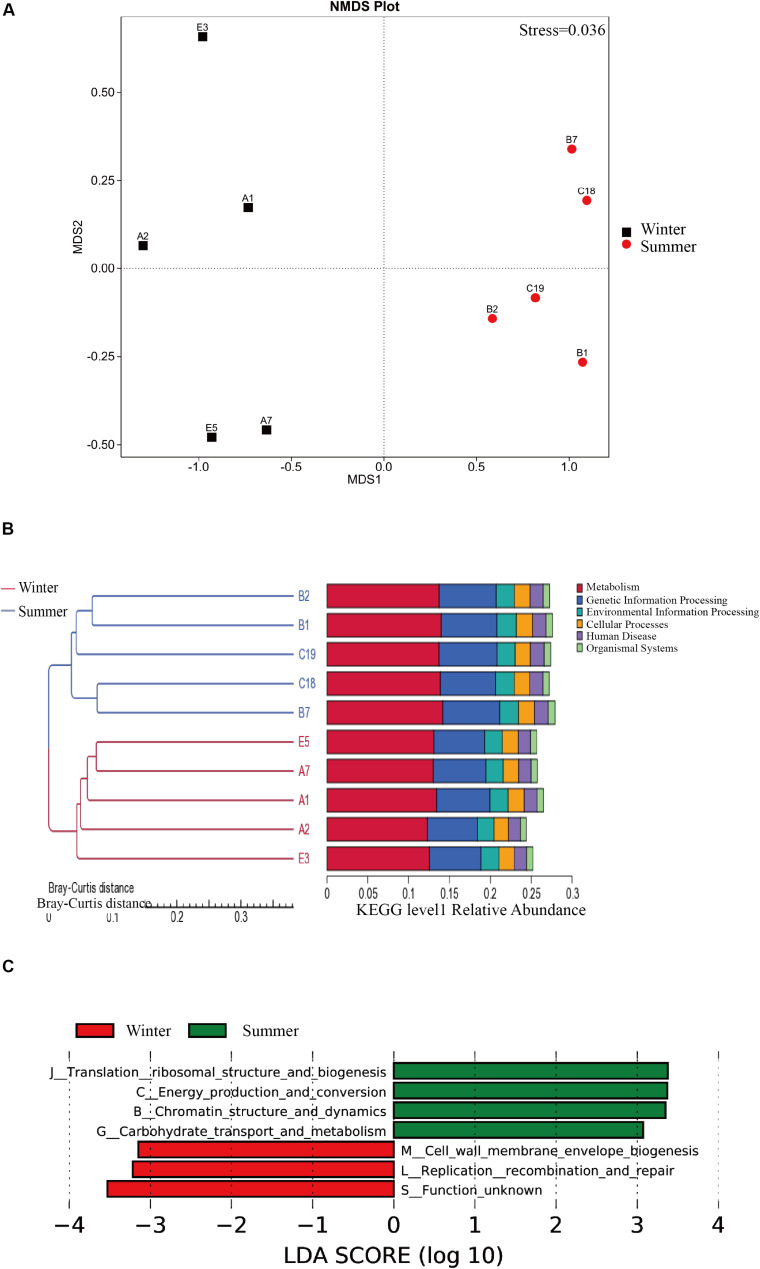
The KEGG pathways differences between summer and winter group. **(A)** The NMDS analysis of metabolic pathways between two seasons based on the KEGG database. **(B)** Heat map of the KEGG level 1 pathways, KEGG database, all the samples were clustered by Bray-Curtis distance. **(C)** Distribution histogram of LDA based on KEGG pathways with statistically significant differences between the summer and winter.

The results from NMDS analysis show that there is a significant different metabolic difference in pathways between the two seasons, and a significant difference in the Kegg function of intestinal flora between summer and winter was found (Figure 3A). The Anosim analysis was used to test the differences between the two groups (Supplementary Figure 2). We used LEfSe (LDA Effect Size) (Score >4) to seek a significant difference in metabolic pathways between summer and winter samples at Kegg level 1 (Figure 3C). Replication recombination and repair are more abundant in the winter group. The following items show significant abundance in summer than winter: carbohydrate transport and metabolism, chromatin structure and dynamics, energy production and conversion, translation ribosomal structure, and biogenesis. We used Metastat analysis to determine top pathways showing significant differences between summer and winter groups (Supplementary Figure 3) and built a clustering heat map based on the 35 functions with the highest abundance (Supplementary Figure 4). The reactions of intestinal flora from the summer group include K03088 (RNA polymerase sigma-70 factor, ECF subfamily), K06147 (ATP-binding cassette, subfamily B, bacterial), K05349 (beta-glucosidase [EC: 3.2.1.21]), K02355 (elongation factor G), K01190 (beta-galactosidase [EC: 3.2.1.23] galactosidase), K03046 (rpoC DNA-directed RNA polymerase subunit beta [EC: 2.7.7.6]), K03043 (rpoB; DNA-directed RNA polymerase subunit beta [EC: 2.7.7.6]), K01006 (ppdK; pyruvate, orthophosphate dikinase), K03737 (pyruvate-ferredoxin/flavodoxin oxidoreductase [EC: 1.2.7.1 1.2.7]), K03497 (ParB family transcriptional regulator, chromosome partitioning protein), and K02469 (DNA gyrase subunit A [EC: 5.6.2.2]). The reactions of intestinal flora from the winter group is K07133 (protein with unknown function) (Supplementary Figure 5).

In addition, the significant enrichment in the SCFAs-yielding pathways was found in the summer group. In total, seven enzymes in the Butyrate metabolism pathways were identified both in the summer and winter groups. All mean that the relative abundance of the enzymes in summer is higher than that in winter. A significant difference among three enzymes (EC:1.8.1.4, EC:1.1.1.157, and EC:2.7.2.7) was found by Wilcox test. Eleven enzymes in the Propionate metabolism pathways were determined in both summer and winter groups. All relative abundance of the enzymes in summer is higher than that in winter, and a significant difference among five enzymes (EC:1.3.5.4, EC:2.8.3.1, EC:6.2.1.17, EC:6.2.1.1, and EC:2.7.2.1) was found by Wilcox test. EC:6.2.1.17 was only found in the summer group (Figure 5, Supplementary Figure 6).

## Discussion

### Seasonal and Sexual Effects on Intestinal Microbial Structure

Male sheep separate from females in the summer, but they converge together in the winter for reproduction purpose, thus, intestinal microbes of the wild blue sheep are characterized by seasonal changes and sexual dimorphism in dietary selection (Figure 3). The variation due to seasonal changes is more prominent than that due to gender differentiation caused by physiology and feeding behaviors. Considering the results of NMDS (Figure 3), the two groups in summer and winter present a significant separation phenomenon, but not between the genders. This shows that sheep maintain very stable intestinal microbial composition in the same season. That is to say, seasonal dietary choices and other external environmental factors play an important role in driving the composition and structure of the intestinal flora.

It is interesting to note that, based on the results from the analysis of alpha diversity, summer males have significantly lower alpha diversity (Figure 1C, Supplementary Table 1). Because of its special relationships with reproduction, special function purposes, and stability, alpha diversity is widely regarded to be an indicator of assessing ecosystem status (Naeem et al., 1994; Kennedy et al., 2002; Isbell et al., 2015; Duffy et al., 2017). It is usually closely related to dietary differentiation. For example, herbivores generally have a higher level of diversity than other mammals (Reese and Dunn, 2018). On the other hand, humans with higher-fiber diets and longer transit time have been found to demonstrate higher-diversity gut microbiota than those living with Westernized lifestyles (Schnorr et al., 2014; Clemente et al., 2015). Furthermore, a diverse diet is expected to create a more metabolic niche for the microbiota, thereby increasing the diversity of the microbial community (Schluter and Foster, 2012). In this study, the diversity of gut microbiota doesn’t show a significant sexual difference in winter, but in summer. Thus, it seems that physiological differences and dietary selection between the sexes in winter won’t influence the microbial diversity of the wild blue sheep, while they are living together, but in summer, while they are separated. In females, there are no seasonal differences in alpha diversity of the gut microbiota. This differentiation, however, exists in males, with a decreased diversity in summer. This implies that seasonal dietary changes are not a determining factor in shaping the micro-environment in sheep’s gut. It seems that a low diversity in males’ gut microbiota during summer is likely to be caused by their own foraging strategy. In our previous research (Chang, 2010), it was indicated that male blue sheep in summer have a larger foraging range than females. They can leave more accessible and higher-quality food to the females who are facing greater nutritional stress in winter of the breeding period, so that males more prefer to take herbaceous plants with a broader distribution range and lower nutrient contents. As a result, the diversity of the recipes in the intestinal microbial community has been reduced in males. This may apply to the cases in the blue sheep analyzed in this study.

We also found that seasonal dietary variation strikingly shapes intestinal flora in summer, which is relatively stable in other seasons. Composition of the intestinal microbiota in the blue sheep is somewhat very similar to that in other ruminants (Costa et al., 2012; Oikonomou et al., 2013; Sun et al., 2020). Firmicutes is the most abundant phylum in blue sheep, followed by Bacteroides. There are also significant seasonal differences: abundant Firmicutes in summer and a significantly higher proportion than in winter (Supplementary Figure 7). Another phylum, Tenericutes, also shows a significant difference between summer and winter (Figure 2) (*W* = 236, *p* < 0.01). A study on the bison indicated that the abundant Tenericute increases following the increased seasonal protein intake (Bergmann et al., 2015). In summer and autumn, bison’s diet has a higher protein proportion (Craine and Dybzinski, 2013). A seasonal difference of Tenericutes in the blue sheep may also be caused by the difference in food protein content between summer and winter.

At the genus level, there are significant differences between the two seasons regarding *Anaerotruncus*, *Oscillibacter*, and *Ruminiclostridium* (Supplementary Figure 8), all of them are Firmicutes. They are strikingly abundant in summer than in winter. *Anaerotruncus* is a butyrate producer (Duncan et al., 2002; Louis et al., 2014), and butyrate is the main energy resource for the colon cells (Scott et al., 2008) to maintain normal physiological functions of the intestine. All three genera are responsible to degrade the fibers during the process of forming organic acids and SCFAs (Koh et al., 2016), facilitating the host to digest complex carbohydrates (e.g., cellulose, hemicellulose) (Dehority and Scott, 1967; McAllister et al., 1994) and absorbing more energy. The calories produced by fiber fermentation account for 10% in Western human society. This proportion in ruminant animals is, however, approximately 50–70%, the main part for energy supply (Bergman, 1990).

Referring to the result from LEfSe analysis (Figure 2), biomarkers in the summer group are Clostrida, Clostridium, and Ruminococcaceae, which is one of the two most abundant flora recorded in mammalian intestines. These bacteria can degrade fibers to produce organic acids and SCFAs (Koh et al., 2016), which may play an important role in maintaining intestinal health (Huws et al., 2011), particularly regarding fiber digestion of the herbivores (Jami and Mizrahi, 2012; Bian et al., 2013). These biomarkers probably make a significant contribution to shaping gut microbes in the summer group, a special adaptation to taking the diet with a higher proportion of the fibers.

Our results indicate that microbial structure in the gut of the blue sheep differs seasonally (Figures 1, 3). Blue sheep is one of the seasonal aggregating ruminants; its structure and composition of microbial flora exhibit significant sexual variation during the segregation period. Males and females, however, tend to have very similar microorganisms after they have gathered together. Blue sheep are distributed in alpine region with enormous diversified food resources from season to season. In summer, their dietary components contain forbs, accounting for 70% of the total; with higher dietary fiber contents, leaves of shrubs are composed of 60% in all dietary categories in winter (Liu et al., 2007; Chang, 2010). Consequently, seasonal variations of intestinal microorganisms respond to seasonal dietary variety. This may be a factor facilitating blue sheep to accumulate enough energy before the sexual congregation in winter for mating purposes.

### Metagenomic Function and Energy Intake Differences Between Summer and Winter

**FIGURE 4:**
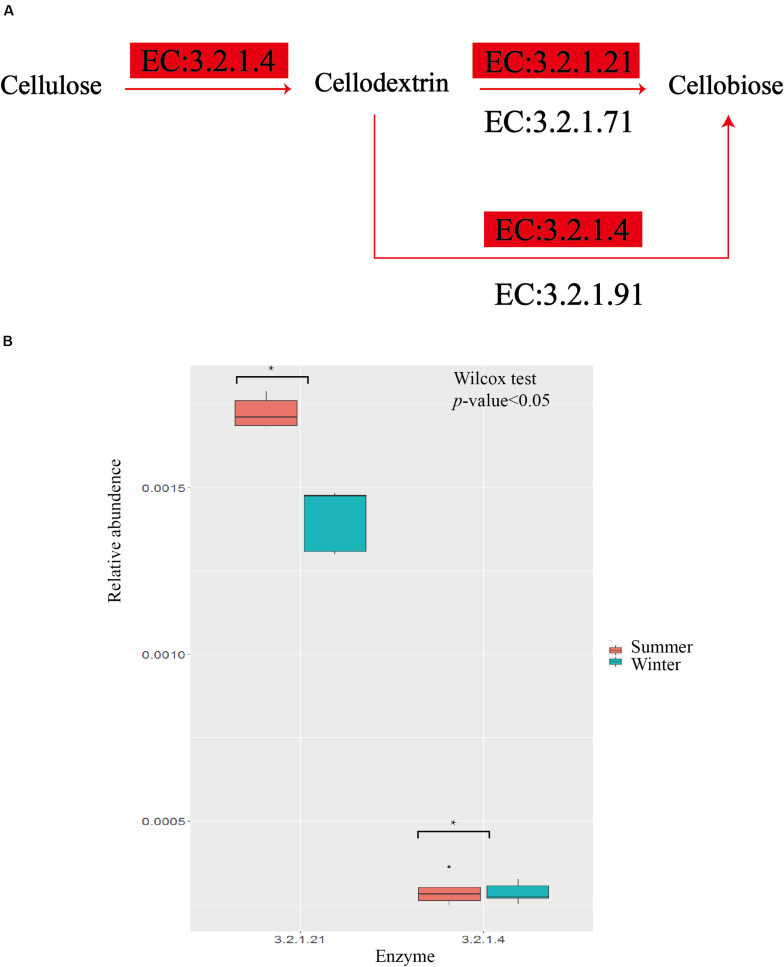
Comparisons of gene and transcript abundance gor enzymes involved in summer and winter. **(A)** The pathway of cellulose degradation, emzymes enriched in summer group are highlighted with red. **(B)** Relative abundence for each enzyme between summer and winter.

**FIGURE 5:**
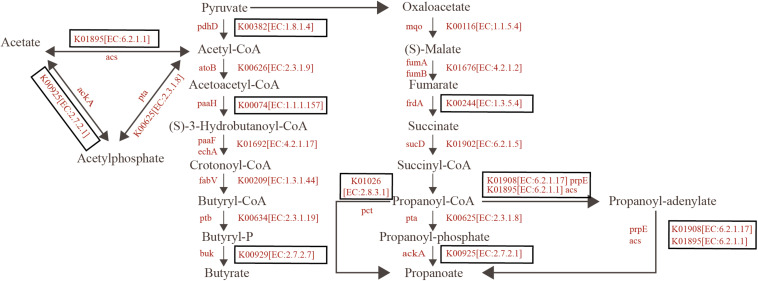
Reconstruction of the metabolic pathways associated with SCAFs formation in the wild blue sheep. KO categories enriched in summer are highlighted with red. Boxed KO categories have statistically significant difference between summer and winter (detailed given in Supplementary Figure 4).

Based on the KEGG pathway database, the summer group has more abundant carbohydrate transport and metabolism pathways, energy production, and conversion pathways (Figure 3C). Within the 12 pathways with the highest abundance between the two groups, 11 of them present more abundance in the summer group, among which K05349, beta-glucosidase [EC: 3.2.1.21], is an important fiber-degrading enzyme. The results from the pathway of cellulose degradation to cellobiose indicate that two important enzymes, EC3.2.1.21 and 3.2.1.4, have significant enrichment in the summer group (Figure 4). Captive populations of the Pere David deer with a higher fiber diet also show a higher proportion of EC3.2.1.21 and 3.2.1.4 enzyme (Wang et al., 2019). The reports on the yak and sheep, another two ruminant mammal species in the Qinghai-Tibet Plateau, indicate that they have higher energy absorption efficiency, and more abundant fiber degradation pathway (Zhang et al., 2016). Except for the advantages of fiber degradation pathways, the summer group also shows enrichment of carbon fixation pathways, for example, the Butyrate metabolism pathways and the Propionate metabolism pathways (Figure 5). This corresponds to the efficient formation of the short-chain fatty acid (Russell and Rychlik, 2001; Fuchs, 2011). Thus, given a higher enrichment of β-glucosidase, and two short-chain fatty acid of the metabolic pathways in the summer group, we speculate that a unique intestinal flora in the summer group helps the host accumulate more energy for the preparation of coming breeding season in winter.

Animal symbiotic microbiota presents a great variation referring to their changeable dietary components and feeding behavioral patterns (Ley et al., 2013; David et al., 2014). Gut microbial flora is also shaped during evolutionary development and by external environmental microbiota (Sullam et al., 2012; Sommer and Baeckhed, 2013; Hale et al., 2018). That blue sheep females and males segregate seasonally can cause gut macrobiotic variation between them because they feed in different habitat niches. Thus, this study indicates that blue sheep have a significant sexual dimorphism in gut microbiota, especially regarding lower intestinal flora diversity due to their different dietary selection and feeding strategy in summer. However, more information on their differences in physiological and nutritional demands is required to interpret such dimorphism mechanism, and their seasonal dietary choices result in significant differentiation in gut microbiota—summer flora helps the hosts to specifically degrade the fibers and produce more short-chain fatty acids and energy. Such a mechanism of natural selection and environmental adaptation allows the blue sheep to hoard much energy, and get ready for the breeding season in winter.

## Data Availability Statement

The datasets GENERATED for this study can be found in NCBI SRA accession PRJNA602195, https://www.ncbi.nlm.nih.gov/bioproject/PRJNA602195.

## Ethics Statement

The animal study was reviewed and approved by The Ethics Committee of Northeast Forestry University.

## Author Contributions

ZZ, YS, SG, and LT conceived and designed the experiments. ZZ, YS, FZ, and ZL carried out the DNA extraction and the data analysis. ZZ, YS, and FZ wrote the manuscript. RP assisted with experiments and advice on manuscript.

## Conflict of Interest

The authors declare that the research was conducted in the absence of any commercial or financial relationships that could be construed as a potential conflict of interest.

## Footnotes

1 http://cutadapt.readthedocs.io/en/stable/

2 https://www.arb-silva.de

3 http://www.drive5.com/usearch/manual/uchime_algo.html

4 http://drive5.com/uparse/

5 http://www.drive5.com/muscle/

6 https://github.com/cjfields/readfq

7 http://bowtie-bio.sourceforge.net/bowtie2/index.shtml

8 https://sourceforge.net/projects/soapdenovo2/files/latest/download?source=files
