# Supplementary material for: Seasonal Variation and Sexual Dimorphism of the Microbiota in Wild Blue Sheep (Pseudois nayaur)
Source: Front Microbiol. 2020 Jun 26;11:1260. doi: 10.3389/fmicb.2020.01260 (PMC7332577; doi:10.3389/fmicb.2020.01260)
Supplement: Supplementary file 1 [file Data_Sheet_1.docx]

**Supplementary Table 1 , Table 2 and Table 3**

Table.1 Comparison of Shannon index of the four groups based on 16S rDNA sequencing.

| Comparison between groups | W value | *p*-value |
| --- | --- | --- |
| SM vs SF | 334.5 | <0.01 |
| SM vs WM | 113 | <0.05 |
| SM vs WF | 326.5 | <0.05 |
| SF vs WM | 216.5 | 0.30 |
| SF vs WF | 256 | 0.23 |
| WM vs WF | 198 | 0.79 |

Table.2 The 5 most abundant phyla in the wild blue sheep microbiota among different groups.

| Taxonomy | SF | SM | WS | WM |
| --- | --- | --- | --- | --- |
| Firmicutes | 61.10% | 57.07% | 49.73% | 51.75% |
| Bacteroides | 26.87% | 33.11% | 31.57% | 31.25% |
| Melainabacteria | 3.23% | 1.88% | 3.98% | 3.12% |
| Tenericutes | 1.27% | 1.16% | 3.18% | 3.38% |
| Euryarchaeota | 0.29% | 0.64% | 2.00% | 2.00% |

Table 3 The Adonis analysis based on the OTUs between the different groups.

| group | Df | SumsOfSqs | MeanSqs | F.Model | R2 | Pr(>F) |
| --- | --- | --- | --- | --- | --- | --- |
| SF_SM | 1(39) | 0.8888(5.3357) | 0.88880(0.13681) | 6.4965 | 0.14279(0.85721) | 0.001 |
| WF_WM | 1(38) | 1.0935(5.7594) | 1.09348(0.15156) | 7.2147 | 0.15956(0.84044) | 0.001 |
