# Supplementary figures and images for: Seasonal Variation and Sexual Dimorphism of the Microbiota in Wild Blue Sheep (Pseudois nayaur)
Source: Front Microbiol. 2020 Jun 26;11:1260. doi: 10.3389/fmicb.2020.01260 (PMC7332577; doi:10.3389/fmicb.2020.01260)

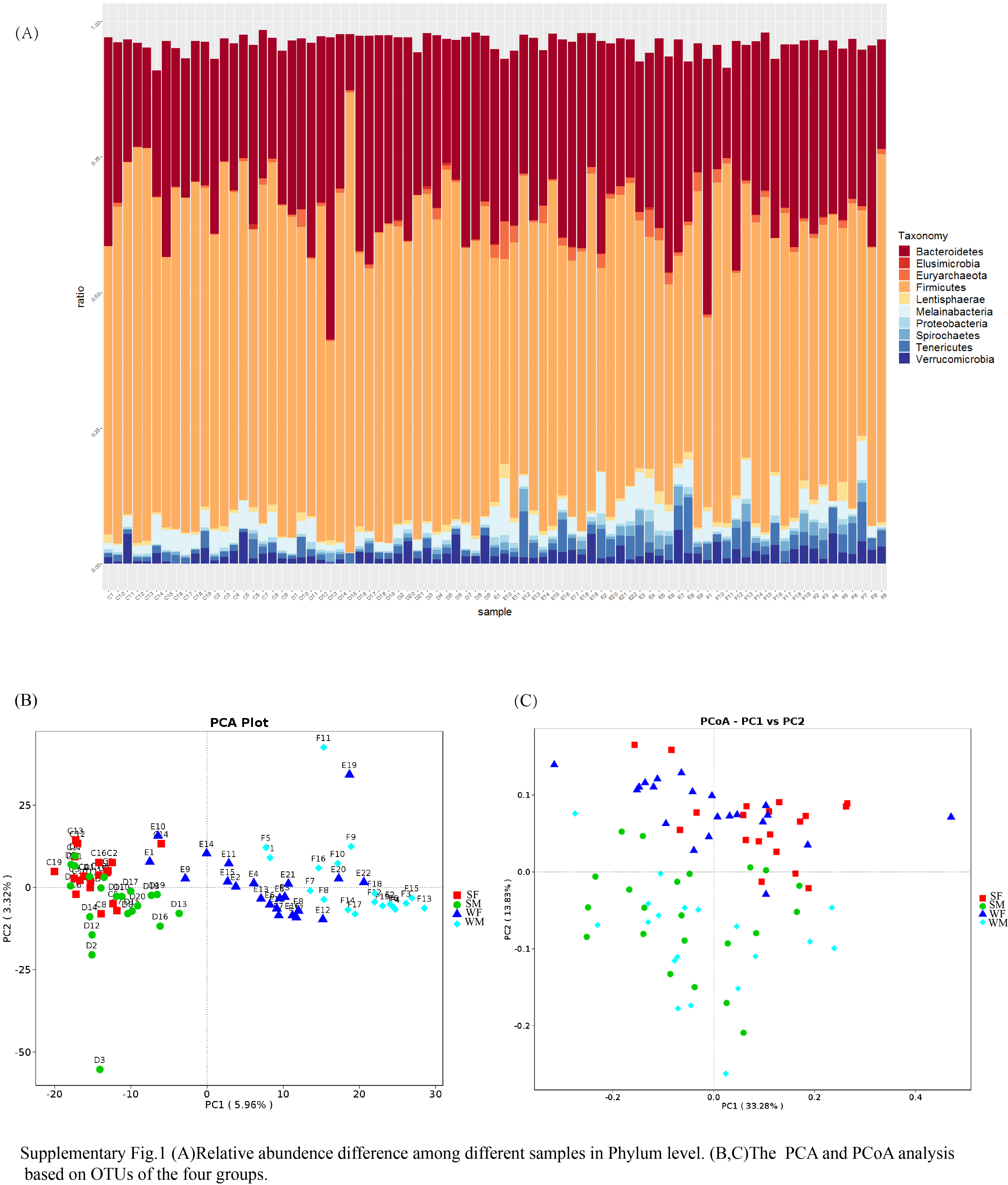

Supplement: Supplementary file 2 [file Image_1.PNG]

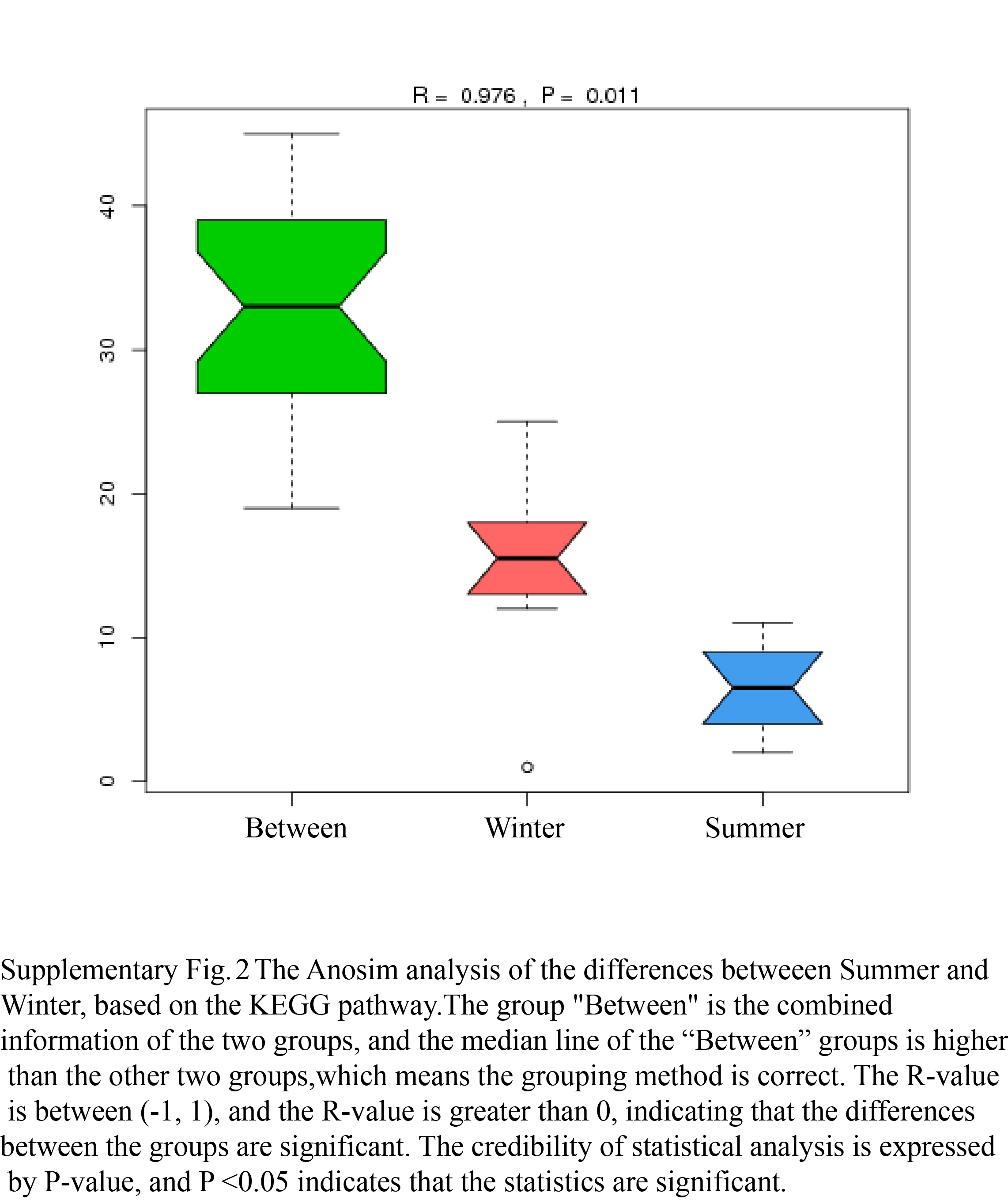

Supplement: Supplementary file 3 [file Image_2.PNG]

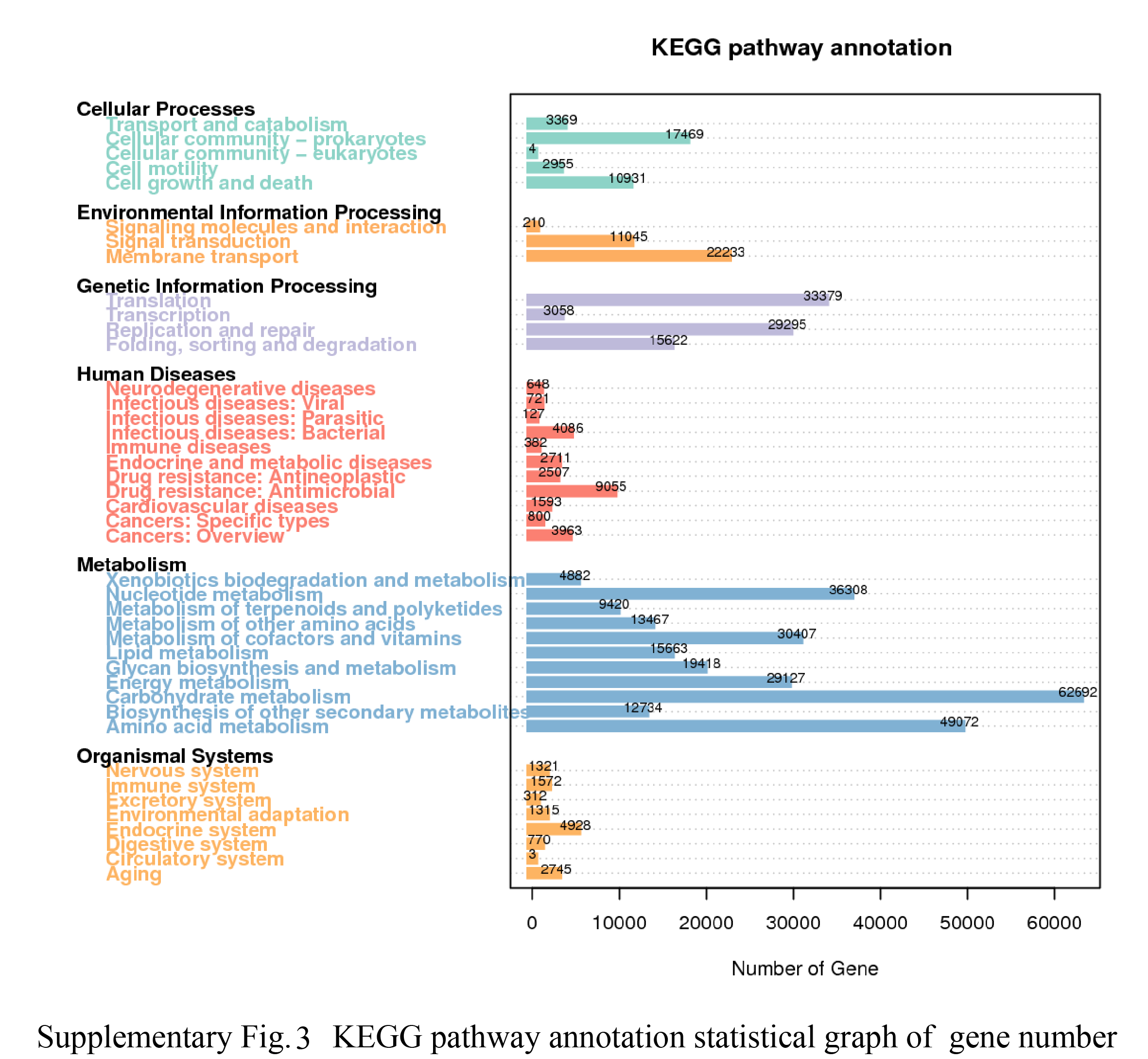

Supplement: Supplementary file 4 [file Image_3.PNG]

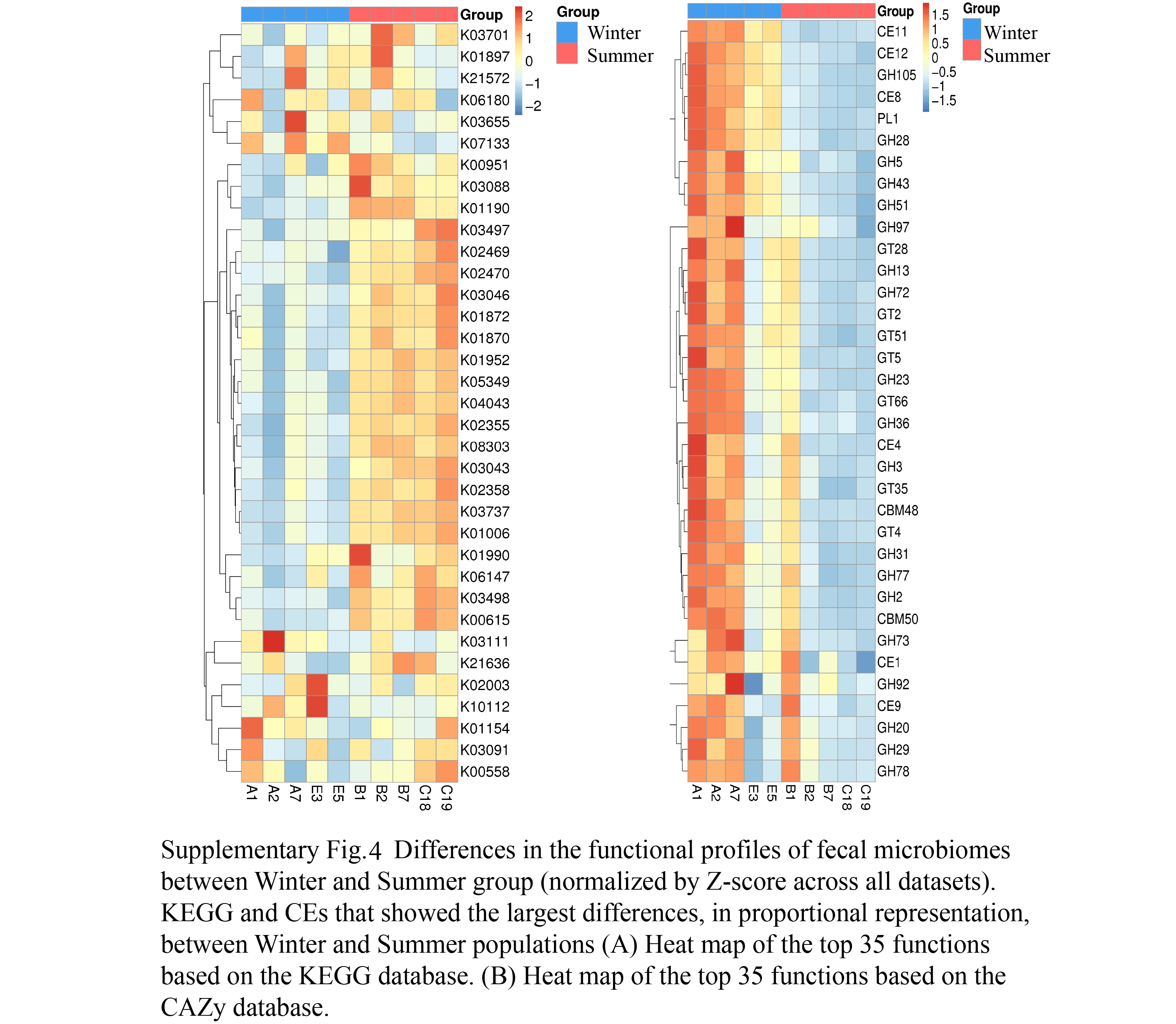

Supplement: Supplementary file 5 [file Image_4.PNG]

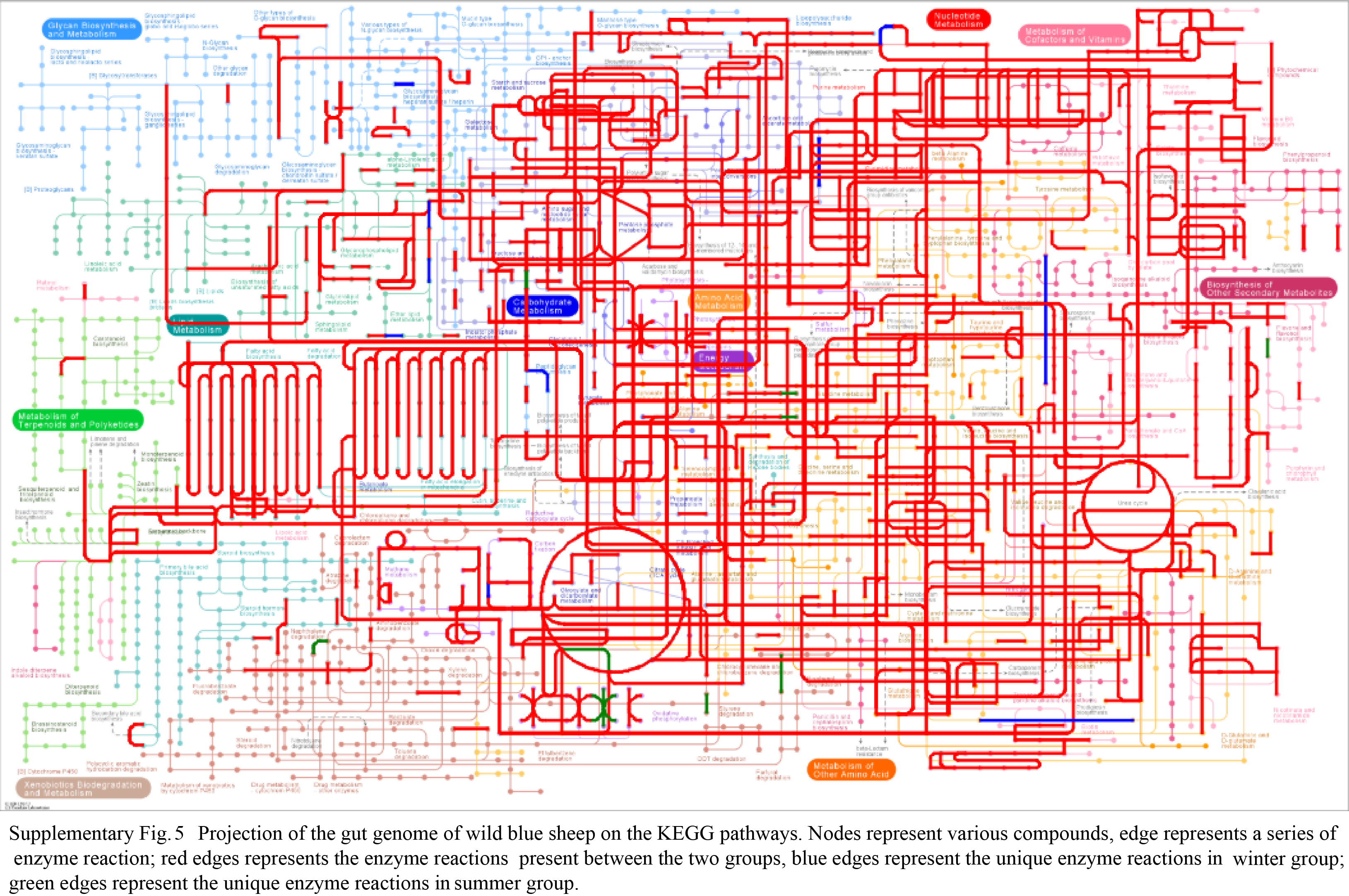

Supplement: Supplementary file 6 [file Image_5.PNG]

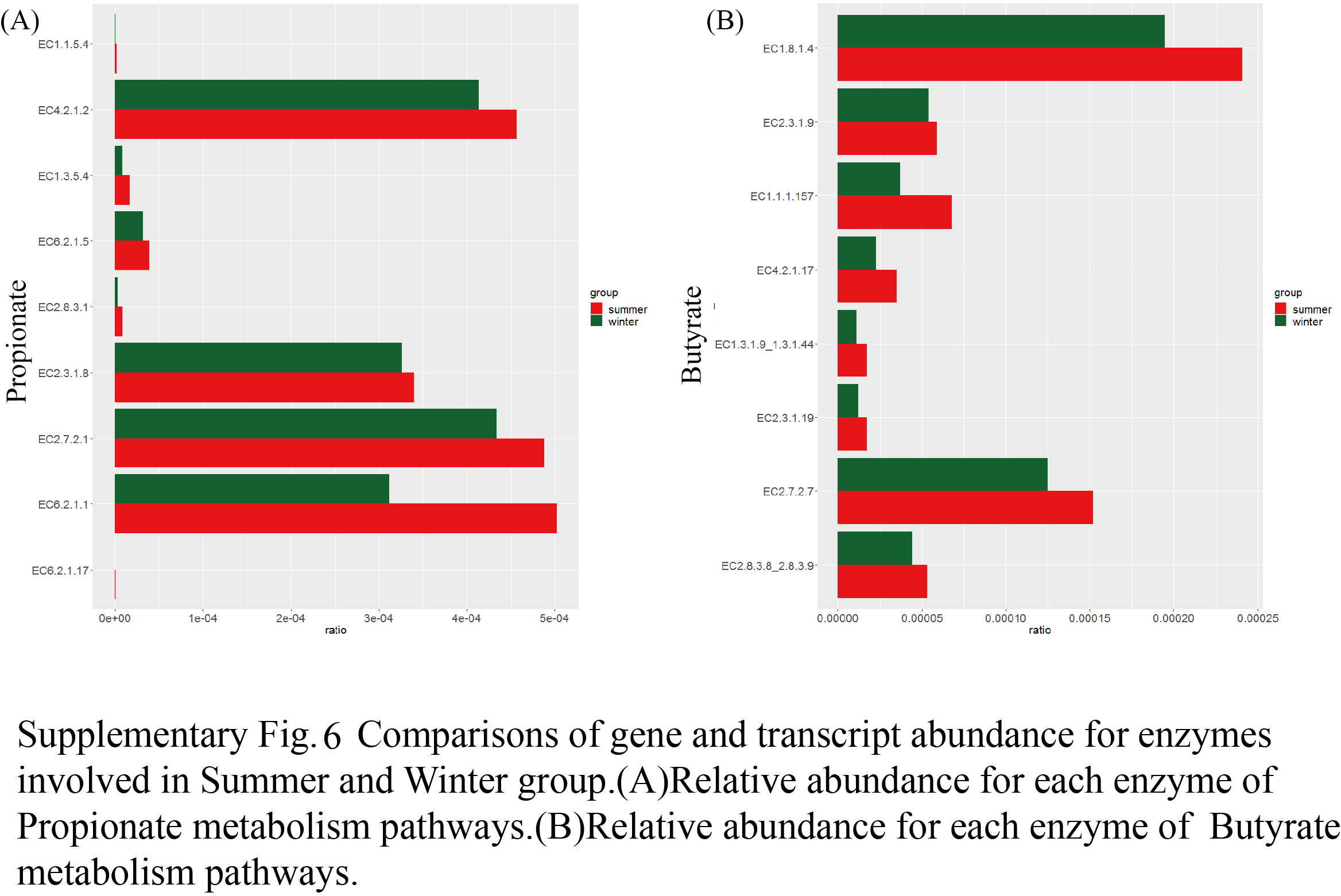

Supplement: Supplementary file 7 [file Image_6.PNG]

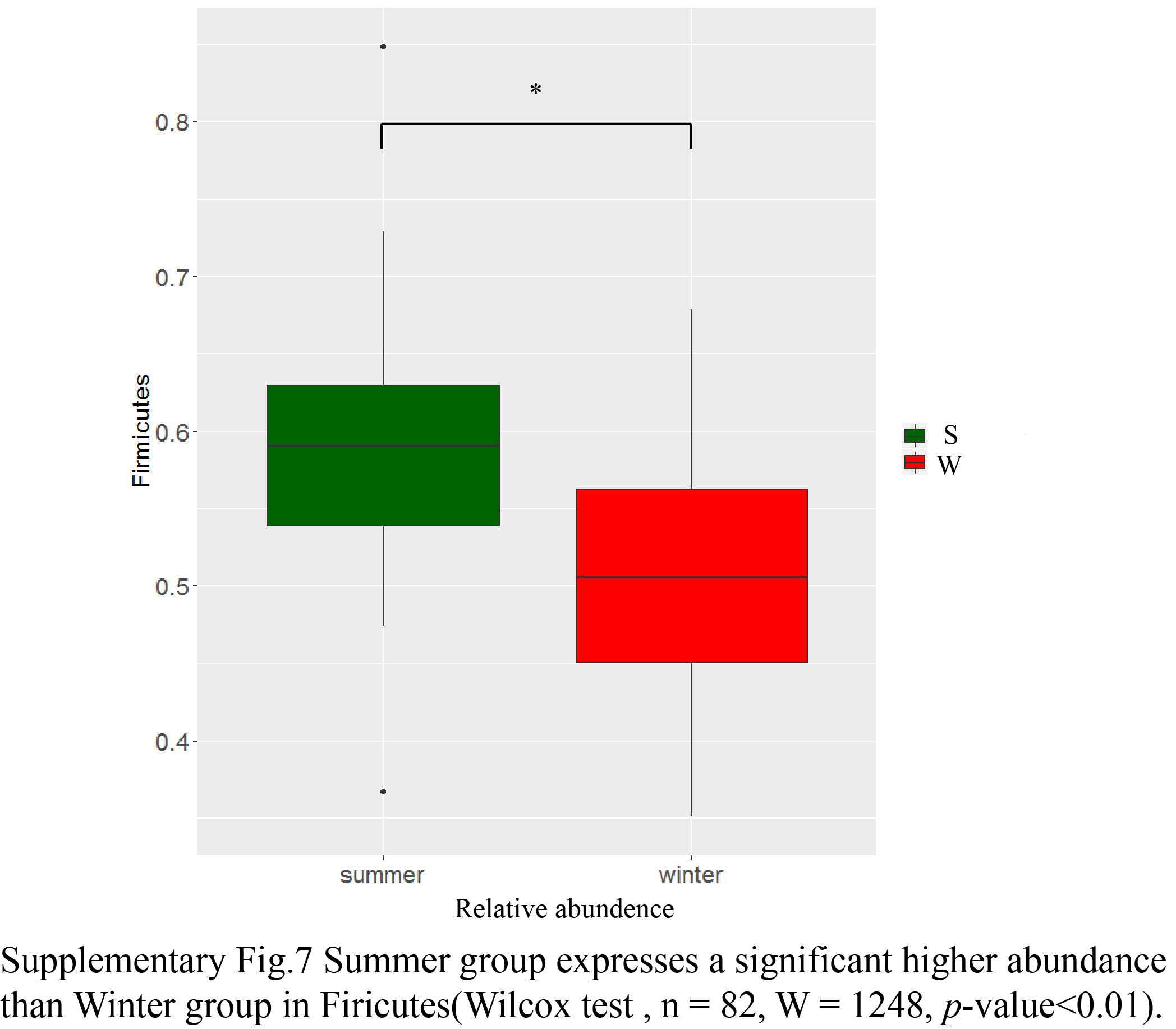

Supplement: Supplementary file 8 [file Image_7.PNG]

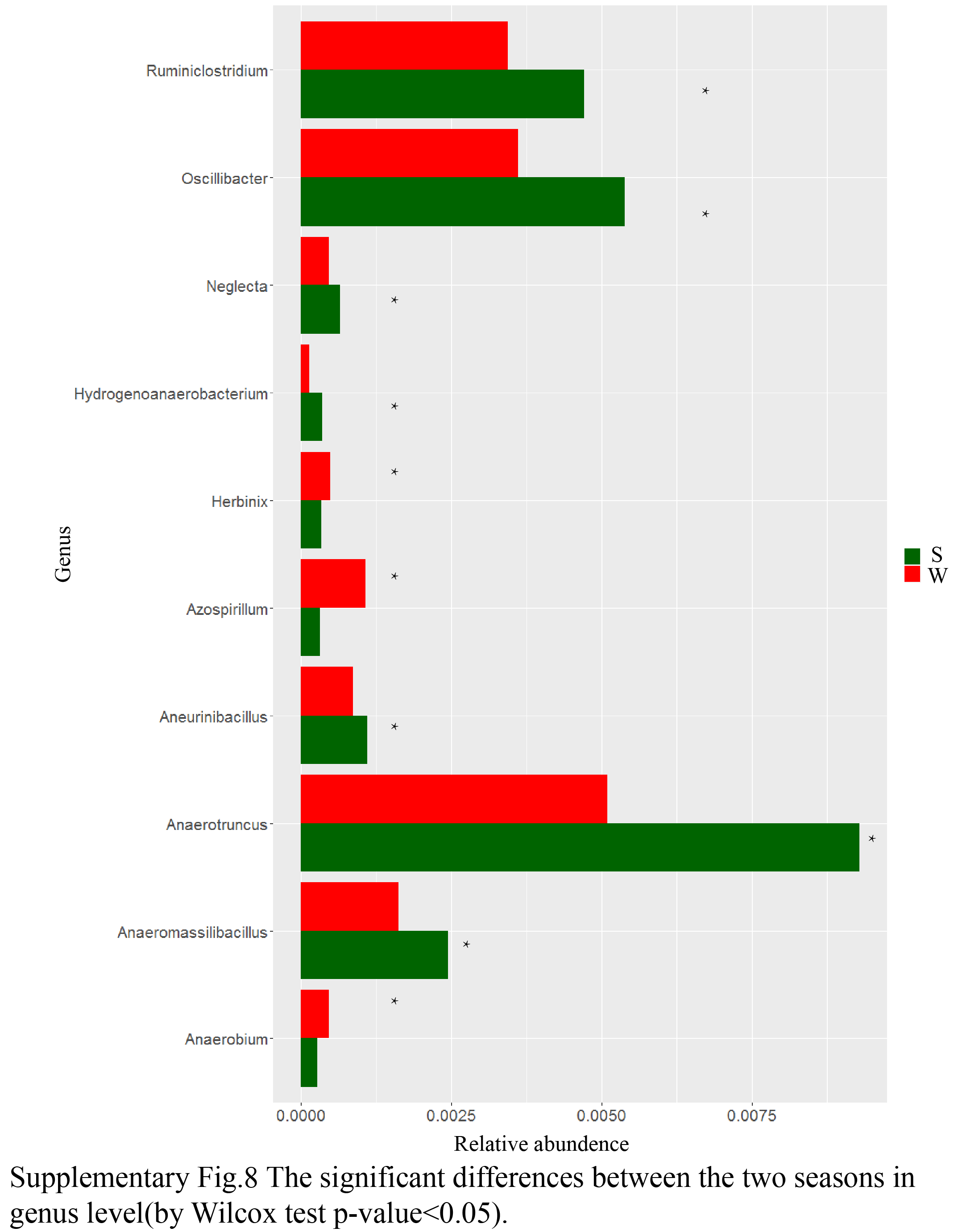

Supplement: Supplementary file 9 [file Image_8.PNG]
